# A Recombinant Protein Based on *Trypanosoma cruzi* P21 Enhances Phagocytosis

**DOI:** 10.1371/journal.pone.0051384

**Published:** 2012-12-10

**Authors:** Adele A. Rodrigues, Tatiana M. Clemente, Marlus A. dos Santos, Fabrício C. Machado, Rafael G. B. Gomes, Heline Hellen T. Moreira, Mário C. Cruz, Paula C. Brígido, Paulo C. F. dos Santos, Flávia A. Martins, Diana Bahia, Juliana T. Maricato, Luiz M. R. Janini, Eduardo H. Reboredo, Renato A. Mortara, Claudio V. da Silva

**Affiliations:** 1 Instituto de Ciências Biomédicas, Universidade Federal de Uberlândia, Uberlândia, Brazil; 2 Departamento de Microbiologia, Imunologia e Parasitologia, Escola Paulista de Medicina - Universidade Federal de São Paulo, São Paulo, Brazil; 3 Instituto de Física de São Carlos, Universidade de São Paulo, São Carlos, Brazil; Louisiana State University, United States of America

## Abstract

**Background:**

P21 is a secreted protein expressed in all developmental stages of *Trypanosoma cruzi.* The aim of this study was to determine the effect of the recombinant protein based on P21 (P21-His_6_) on inflammatory macrophages during phagocytosis.

**Findings:**

Our results showed that P21-His_6_ acts as a phagocytosis inducer by binding to CXCR4 chemokine receptor and activating actin polymerization in a way dependent onthe PI3-kinase signaling pathway.

**Conclusions:**

Thus, our results shed light on the notion that native P21 is a component related to *T. cruzi* evasion from the immune response and that CXCR4 may be involved in phagocytosis. P21-His_6_ represents an important experimental control tool to study phagocytosis signaling pathways of different intracellular parasites and particles.

## Introduction

Phagocytosis is the way by which macrophages internalize foreign particles and cellular debris. A diverse repertoire of receptors expressed by macrophages allows them to respond to a variety of external stimuli. Immune receptors come in a variety of classes, such as the well-characterized phagocytic Fcγ receptor (FcγR) and the complement receptor (CR). Much of our understanding of phagocytic signaling comes from studies on FcγR-mediated phagocytosis, which is a spatially and temporally coordinated series of events initiated by the binding of an opsonized IgG particle with FcγR that leads to actin polymerization and the formation of pseudopods that extend around the particle to form a phagocytic cup. The pseudopods then completely surround the particle forming a phagosome, which is then internalized and its contents degraded (for review: [1]).

To promote phagocytosis, macrophages must be recruited, where chemokines play a crucial role during this process. Nonetheless, the signaling pathways for the various chemokines, leading to efficient chemotaxis of macrophages, are not yet well characterized [1]. Chemokines act through the G protein coupled receptor (GPCR) superfamily, such as CCR5 and CXCR4. Ligand binding to GPCRs induces conformational changes of the receptor that are transmitted to the cytoplasmic domains of the protein, enabling the protein to couple with an intracellular heterotrimeric G protein. The intracellular G protein acts as an intracellular signal by activating or inhibiting cytoplasmic enzymes. CCR5 was first isolated as a functional GPCR that is antagonized by various CC chemokines. CXCR4 was originally identified as an orphan receptor called leukocyte-derived seven transmembrane domain receptor (LESTR), but did not receive much attention until its isolation as a coreceptor for HIV-1 and the discovery of its natural ligand, SDF-1/CXCL12. In their function as HIV coreceptors, CCR5 and CXCR4 physically associate with CD4-activated gp120, which undergoes a conformational change that in turn exposes a hidden coreceptor-binding site. Binding of gp120 to the coreceptor brings the envelope into close proximity to the cell surface and induces gp120 to undergo a second conformational change that allows the gp41 protein to penetrate the cell membrane and form a six-helix array. Through processes that are still unknown, fusion occurs between the cell and viral membranes allowing entry of the viral capsid and proteins (for review: [2]).

F-actin is required for CD4 and CXCR4 redistribution, and it has been shown that activated moesin promotes F-actin redistribution and CD4-CXCR4 clustering, which are required for efficient X4-tropic HIV-1 infection in permissive lymphocytes [3]. Also, authors have proposed that Arf6 (ADP ribosylation factor 6) GTP/GDP activity has synergy with the key first HIV-1/receptor interactions by maintaining PIP_2_-associated membrane dynamics to promote efficient viral fusion and entry in a clathrin-independent manner [4]. Moreover, CXCR4 and CD74 lead to macrophage migration inhibitory factor (MIF) endocytosis [5], and RpkA, a highly conserved GPCR of *Dictyostelium discoideum,* plays a role in phagocytosis and anti-bacterial defense [6]. Thus, these data suggest that GPCRmay play important role during phagocytosis in addition to the traditionally well-described functions related to chemotaxis, adhesion, cell survival and proliferation. Moreover, it has been shown that the uptake of *Yersinia pestis* by macrophages lacking CCR5 is significantly decreased [7].

*Trypanosoma cruzi* can infect and replicate in macrophages. During invasion, *T. cruzi* interacts with different macrophage receptors to induce its own phagocytosis. However, the nature of those receptors and the molecular mechanisms involved are poorly understood. The phagocytosis of tissue culture-derived trypomastigotes (TCT) is mediated by macrophage Pronase-sensitive membrane components. Also, there is indirect evidence that FcγR and CR participate in *T. cruzi* phagocytosis but are not essential [8]. Moreover, *T. cruzi* amastigote forms interact with both the macrophage mannose receptor and mannose-binding proteins in a way that facilitates the adhesion of amastigotes to macrophages [9]. Recently, authors have shown that phagocytosis induced by *T. cruzi* infection involves Toll-like receptor (TLR) 2 but are independent of TLR4 receptors [10]. In addition, a previous report suggested that once the surface components of *T. cruzi* trypomastigotes are recognized by macrophage receptors, they trigger the activation of a tyrosine phosphorylation cascade, PI3-kinase recruitment, and assembly of actin filaments at the site of initial cell-to-cell contact, resembling the events described during phagocytosis [11].

We have recently characterized a novel *T. cruzi* protein whose role during parasite cell invasion is not completely understood. First, we searched *T. cruzi* genomic database for species-specific hypothetical proteins that showed a high probability of being secreted or membrane-anchored and thus likely to be involved in host-cell invasion. A sequence that codes for a 21-kDa protein with a high probability of being secreted was selected. After cloning this protein sequence, we found that it was a ubiquitous secreted protein of *T*. *cruzi*. The recombinant form (P21-His_6_) adhered to HeLa cells in a dose-dependent manner. Also, HeLa cell treatment with P21-His_6_ augmented invasion by *T. cruzi* metacyclic trypomastigotes and extracellular amastigotes [12]. Since P21 promoted *T. cruzi* cell invasion, we examined its potential to enhance the phagocytosis of zymosan particles and protozoan parasites by mouse inflammatory macrophages. Our results suggested that P21-His_6_ binds to CXCR4 expressed in macrophages and acts as a phagocytosis inducer by activating actin polymerization in a way dependent on the PI3-kinase signaling pathway. This is the first study that shows a possible role of CXCR4 in mediating phagocytosis and that P21-His_6_ may represent an important experimental tool to elucidate the signaling pathway for the phagocytosis of particles and intracellular parasites.

## Results

### P21-His_6_ Upregulates Zymosan Phagocytosis

**Figure 1:**
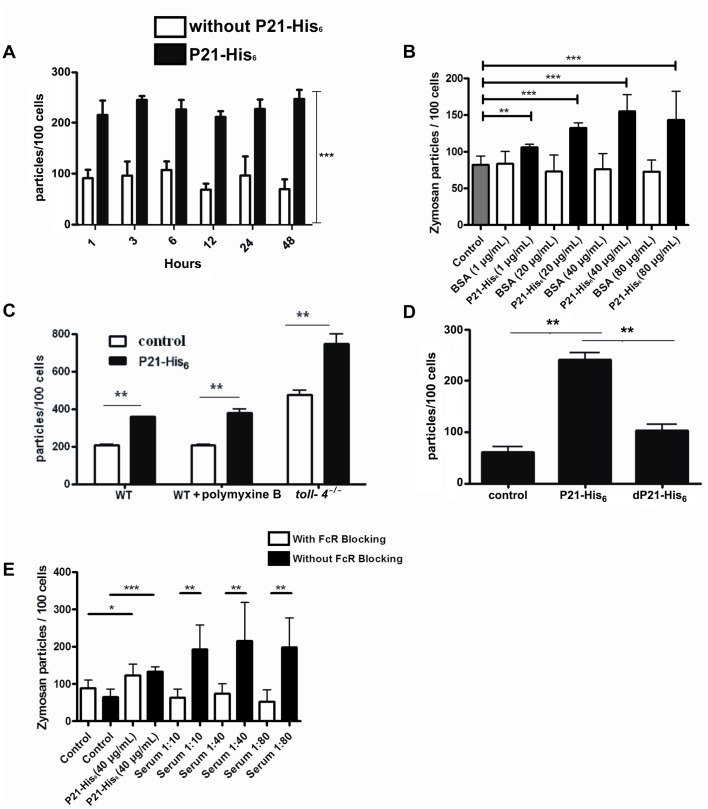
P21-His_6_ enhances phagocytosis of zymosan particles. Phagocytosis assay using zymosan particles treated or not with P21-His_6_. (A) peritoneal macrophages, treated in different periods,1, 3, 6, 12, 24 and 48 hours. P21-His_6_ enhanced zymosan internalization, in all treatment periods. (B) Peritoneal macrophages (grey bar), treated with BSA (white bars) or P21-His6 (black bars) at different concentrations: 1, 20, 40 and 80 µg/ml. P21-His6, but not BSA, enhanced zymosan internalization at all concentrations tested. (C) Peritoneal macrophages obtained from wild-type (WT) or *toll4^−/−^* animals, treated or not with P21-His_6_, and those also treated with polymyxin B. P21-His_6_ treatment increased zymosan phagocytosis in both, the WT and *toll4^−/−^* macrophages. Also, treatment with polymyxin B did not inhibit P21-His_6_ activity. (D) Phagocytosis assay using folded and denatured P21-His_6_ (dP21-His_6_) at 100°C. Note that only folded protein was able to upregulate phagocytosis. (E) Peritoneal macrophages with (white bars) or without (black bars) FcR blocked were incubated with P21-His_6_ (40 µg/ml) previously opsonized in different concentrations of serum. Peritoneal macrophages with FcR did not show an enhanced zymosan internalization as those without FcR blocked. *P<0.05 **P<0.01 ***P<0.001.

To determine if P21-His_6_ would upregulate phagocytosis, we incubated C57BL/6 peritoneal macrophages with different concentrations of P21-His_6_ and zymosan particles. We also tested 40 µg/ml of the recombinant protein at different time points. P21-His_6_ increased phagocytosis in a dose- and time- independent manner. Incubation with BSA showed no impact on macrophage phagocytosis activity (Figure 1A, B). Since P21-His_6_ was purified from *Escherichia coli*, we needed to ascertain that the observed phenotype was due to the recombinant protein activity *per se* and not to residual bacterial LPS. Thus, we performed the zymosan phagocytosis assay in the presence of polymyxin B, using macrophages derived from *toll-4^−/−^* mice. The results showed that the pro-phagocytic activity observed was in fact due to the effect of P21-His_6_ on the macrophages (Figure 1C). Also, we performed similar uptake experiments using denatured P21-His_6_, which showed a significantly lower zymosan uptake (Figure 1D). In addition, previous incubation of the recombinant protein with anti-P21-His_6_ polyclonal antibody decreased phagocytosis to basal levels in FcR-blocked macrophages (Figure 1E). On the other hand, macrophages without FcR blocking also showed high phagocytosis rates.Pre-immune serum sample at the same dilutions as anti-P21-His_6_ did not affect P21-His_6_ activity (data not shown).

### P21-His_6_ Induces Macrophage Actin Polymerization

**Figure 2:**
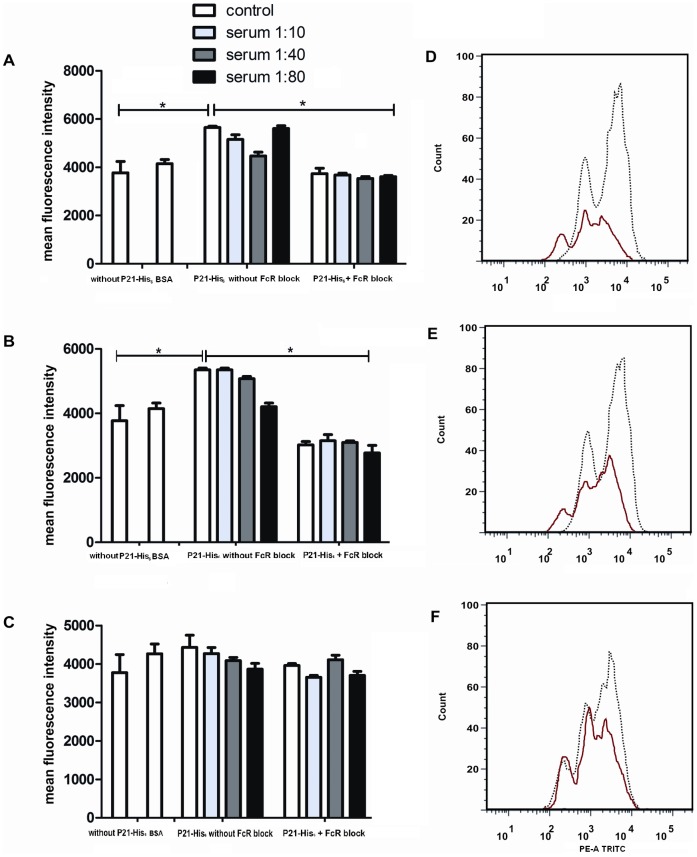
Peritoneal macrophages treated with P21-His_6_ show increased actin polymerization. Peritoneal macrophages were treated or not with P21-His_6_ for 0.5, 1.0 and 3.0 hours in the presence or not of anti-P21-His_6_ polyclonal antibody and FcR blocking. The actin cytoskeleton was stained with TRITC-phalloidin. Flow cytometry showed that the cells treated with the recombinant protein had a higher fluorescence intensity than the control cells following 0.5 (A) and 1.0 hour (B) of incubation. After 3.0 hours of incubation, the protein lost its activity (C). Representative histograms are also shown. Macrophages not treated with P21-His_6_ (D); macrophages treated with P21-His_6_ (E); macrophages with FcR-blocking and treated with P21-His_6_ previously incubated with anti-P21-His_6_ polyclonal antibody (F).*P<0.5, **P<0.01, ***P<0.001.

Phagocytosis is a process dependent on actin polymerization. Hence, peritoneal macrophages wereincubated with 40 µg/ml of P21-His_6_ for 30 minutes and 1 and 2 hours and afterwards stained with TRITC-phalloidin. Cell samples were then analyzed by flow cytometry to determine the status of actin polymerization by measuring fluorescence intensity. Actin polymerization was increased as assessed by the intensity of phalloidin labeling of treated cells when compared to untreated controlmacrophages (Figure 2 A, B).Also, anti-P21-His_6_ polyclonal antibody abolished actin polymerization only in FcR-blocked macrophages (Figure 2 A, B). After 3 hours of treatment, the recombinant protein lost its activity (Figure 2 C). A pre-immune serum sample at the same dilutions as anti-P21-His_6_ did not affect P21-His_6_ activity (data not shown).

### P21-His_6_ Regulates Phagocytosis and Actin Polymerization by Activating the PI3-kinase Signaling Pathway

**Figure 3:**
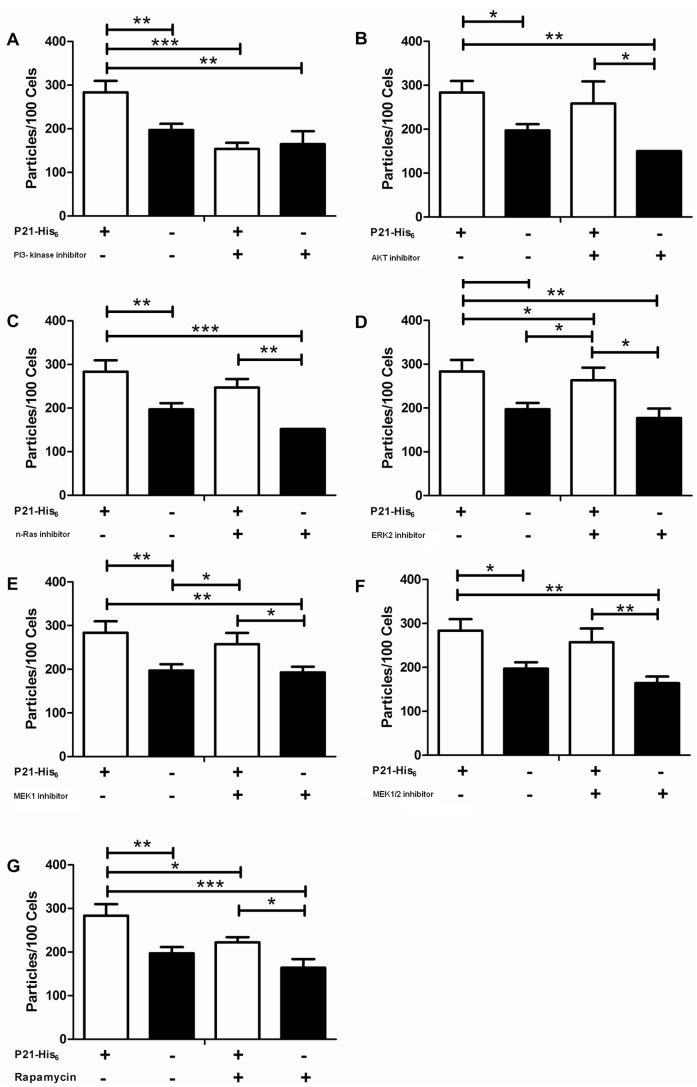
P21-His_6_ activity depends on PI3-kinase pathway. Peritoneal macrophages were incubated with zymosan, treated or not with P21-His_6_, and also treated or not with inhibitors to PI3-kinase (A), AKT (B), n-Ras (C), ERK 2 (D), MEK 1 (E), MEK1/2 (F), m-Tor (G). P21-His_6_ activity was not detected in the presence of PI3-K inhibitor. *P<0.5, **P<0.01, ***P<0.001.

Phagocytosis assays in the presence or absence ofP21-His_6_as well as different signaling pathway inhibitors showed that the pro-phagocytic activity of P21-His_6_ depended on the PI3-kinase signaling pathway (Figure 3 A). The recombinant protein did not show pro-phagocytic activity in macrophages treated with PI3-kinase inhibitor. Although a small statistically significant difference was observed for macrophages treated with m-Tor and ERK 2 inhibitors, the recombinant protein still showed high pro-phagocytic activity almost reaching its performance rates compared to untreated control macrophages. Thus, the statistical significance of the effects observed for the m-Tor and ERK2 signaling pathways did not seem to be correlated to a major biological impact on pro-phagocytic property of P21-His_6_ (Figure 3 D, G). Moreover, in macrophages treated with AKT, n-Ras, MEK 1 or MEK1/2, P21-His_6_ induced phagocytosis to a similar extent as the untreated control cells (Figure 3 B, C, E, F).

### P21-His_6_ Binds to CXCR4 Chemokine Receptor

**Figure 4:**
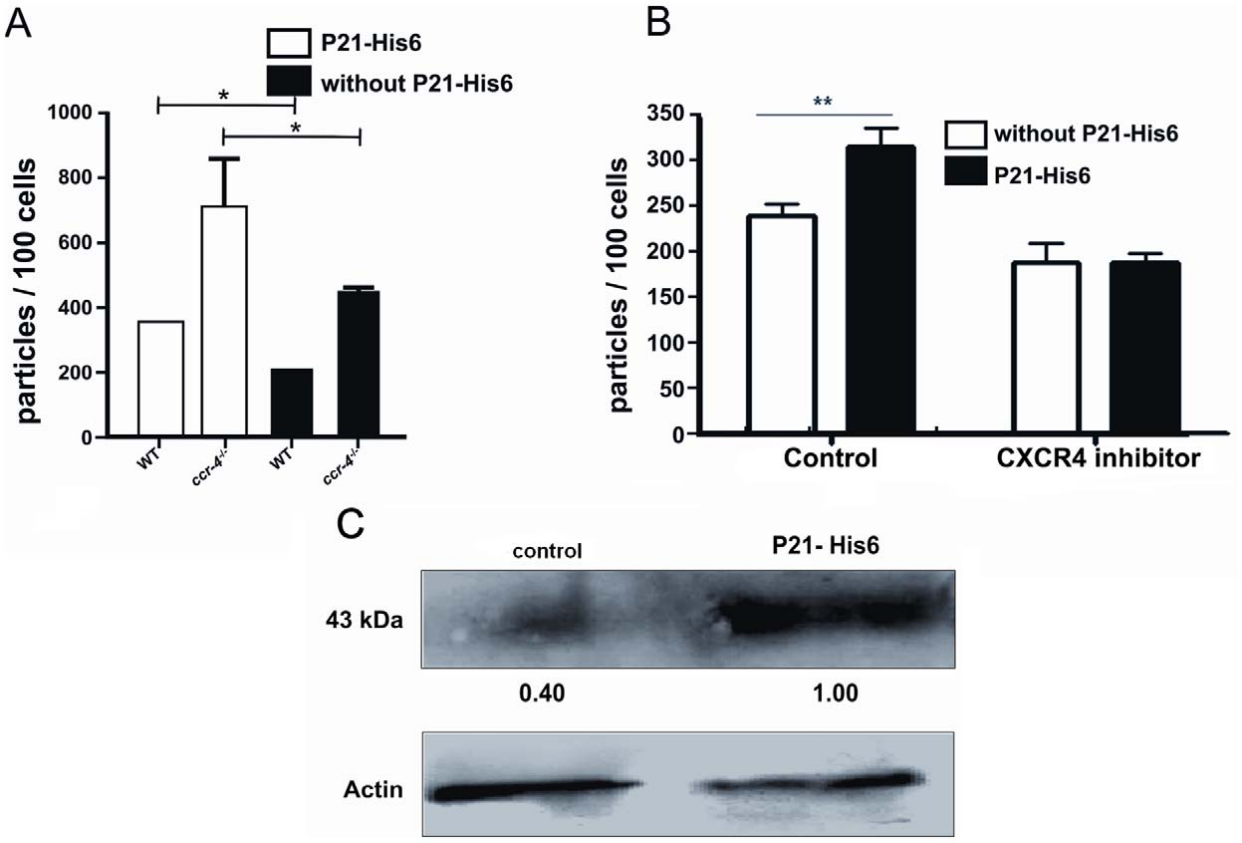
P21-His_6_ binds to CXCR4 chemokine receptor. CCR4 knockout did not affect P21-His_6_ pro-phagocytic activity (A). CXCR4 inhibitor abolished the pro-phagocytic P21-His_6_ activity (B). Peritoneal macrophages treated with P21-His_6_ showed an increased expression of CXCR4 revealed by Western blotting (C). *P<0.5, **P<0.01, ***P<0.001.

We hypothesized that a GPCR would be the mammalian cell receptor for the protein. To gain insight into this aspect, we performed the zymosan phagocytosis assay using macrophages treated or not with AMD3100 (CXCR4 inhibitor) and macrophages from *ccr4^−/−^*mice in the presence or absence of P21-His_6_. Host cell treatment with CXCR4 inhibitor completely abolished P21-His_6_ pro-phagocytic activity (Figure 4 A, B) indicating CXCR4 could be the macrophage receptor for P21-His_6_. We also performed Western blotting of peritoneal macrophages treated or not with P21-His_6_to determine the amount of CXCR4 expression. Band densitometry showed that CXCR4 expression was increased in macrophages treated with P21-His_6_ (Figure 4 C). Taken together, these results strengthened the idea that the receptor for the recombinant protein in macrophages is CXCR4.

### P21-His_6_ Increases Phagocytosis of Different Intracellular Parasites

**Figure 5:**
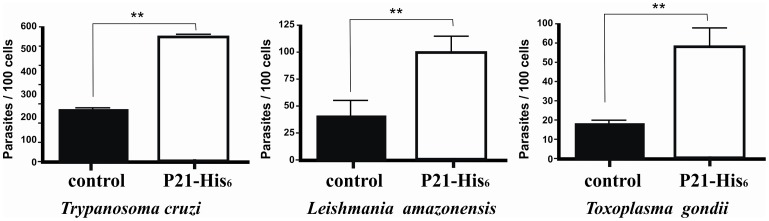
P21-His_6_ augments phagocytosis of different intracellular parasites. Peritoneal macrophages were incubated with *Trypanosoma cruzi*, *Leishmania amazonensis* or *Toxoplasma gondii* and treated or not with P21-His_6_. The number of internalized parasites in treated cells was higher than in control cells, indicating that P21-His_6_ increases parasite internalization. *P<0.5, **P<0.01, ***P<0.001.

Finally, we turned our attention to the possible effect of the recombinant protein on *Leishmania amazonensis* and *Toxoplasma gondii* uptake by macrophages. Here, we used *T. cruzi* extracellular amastigotes as the positive control [12]. The results obtained confirmed our hypothesis that P21-His_6_ upregulates phagocytosis by macrophages. In macrophages treated with P21-His_6_, there was a threefold increase in the phagocytosis of *T. cruzi* extracellular amastigotes and also a fourfold and fivefold increase in the uptake of *L. amazonensis* promastigotes and *T. gondii* tachyzoites, respectively (Figure 5).

## Discussion

Endocytosis is essential for virtually all eukaryotic cells to internalize nutrients, antigens, pathogens, and cell surface receptors from the plasma membrane into membrane-bounded, endocytic vesicles to regulate cell homeostasis, cell signaling, and development. Distinct mechanisms mediate endocytic uptake of a large variety of different-sized cargoes ranging from small molecules to viruses or bacteria [13]. In this context, macrophages are strategically located throughout body tissues, where they ingest and process foreign materials, dead cells and debris and recruit additional macrophages in response to inflammatory signals [14].Moreover, authors have identified a small GTPase effector protein, IQGAP1, as a novel diacylglycerol kinase (DGKζ)-associated complex protein, and DGKζhas been found to be involved in IQGAP1/Rac1-mediated bacterialphagocytosis upon LPS stimulation in macrophages [15].

Here we evaluated the potential of a recombinant protein based on *T. cruzi* P21 in upregulating phagocytosis, its putative macrophage receptor and the signaling pathway triggered upon P21-His_6_ interaction with the host cell.Our results showed that P21-His_6_ is able to upregulate phagocytosis in a way dependent on binding to CXCR4 actin polymerization and the signaling pathway triggered by PI3-kinase.

P21-His_6_ pro-phagocytic activity depended neither on time of incubation nor protein concentration. Also, polyclonal antibody anti-P21-His_6_, denaturated protein, polymyxin treatment and the use of peritoneal macrophages derived from *toll4^−/−^* mice confirmed that the pro-phagocytic activity observed in different assays was due to the recombinant protein and not to residual LPS from the bacterial extract. Experiments performed without FcR blocking showed an increased phagocytosis probably due to the cross-linking of FcR by the immune complex.

Actin assembly during phagocytosiscreates protrusions that encompass extracellular materials. Actin also supports the processes of invagination of a membrane segment into the cytoplasm, elongation of the invagination, scission of the new vesicle from the plasma membrane, and movement of the vesicle away from the membrane [16]. Accordingly, macrophage actin polymerization triggered by P21-His_6_ was demonstrated by flow cytometry. The results supported our findings, since P21-His_6_ triggered actin polymerization at the time points studied and since this activity was completely abolished following treatment with anti-P21-His_6_ and FcR blocking of macrophages.

Turning our attention to the putative signaling pathway triggered upon P21-His_6_ interaction with peritoneal macrophages, our results using inhibitors of different signaling pathways showed that P21-His_6_ relies on the PI3-kinase signaling pathway in a way independentof AKT. This intriguing result is supported by other studies [17], [18]. Studies have demonstrated that PI3K-dependent signal transduction of Rho-family GTPase activities occurs during FcR-mediated phagocytosis and that PI3K-dependent deactivation of Cdc42 is necessary for phagocytosis. Moreover, the activities of PI3K and Cdc42 are linked: FcR-activated Cdc42 stimulates PI3K, which increases concentrations of PI(3,4,5)P_3_ in phagocytic cups, allowing the PI(3,4,5)P_3_-dependent deactivation of Cdc42 that is necessary for completion of phagocytosis [19]. In addition, a study has provided important evidence on PI3-kinase activation during *T. cruzi* cell invasion [20]. Also, we observed a small statistical difference during zymosan phagocytosis by macrophages that were previously treated with m-Tor and ERK2 inhibitors in the presence of P21-His_6_. Besides, the recombinant protein showed high pro-phagocytic activity. Thus, these results indicated that these signaling pathways may not be too relevant for the biological process triggered by P21-His_6_. We have a study underway to better understand P21-His_6_ signaling process, using besides inhibitors, other approaches such as Western blotting of phosphorylated proteins, PCR array and phosflow kits.

Considering the variety of cell surface receptors, we hypothesized that GPCRs could be the host cell receptor for P21-His_6_ according to recently published studies [6], [7]. We performed experiments using peritoneal macrophages from *ccr4^−/−^*mice and using CXCR4 inhibitor. Our results from experiments using CXCR4 inhibitor showed that CXCR4 could be the P21-His_6_ receptor in macrophages. As a GPCR, the mechanism of CXCR4 receptor activation is mediated by coupling to an intracellular heterotrimeric G-protein associated with the inner surface of the plasma membrane [21], [22]. CXCR4 signaling during chemotaxis, adhesion, cell survival and proliferation has been shown to involve PI3-kinase, AKT, MAPK, the Ras-activated signaling pathway and mammalian target of rapamycin complex 1 (mTORC1) [22]–[25]. On the other hand, the signaling pathway triggered during CXCR4-dependent phagocytosis is largely unknown, since this is the first report to consider CXCR4 as a phagocytosis mediating receptor. This issue will be further investigated in novel studies from our group.

Recombinant DNA technology has allowed scientists to prepare large amounts of proteins based on their native ones to study the putative activity of the native protein. This study revealed that P21 may be an important *T. cruzi* host immune response evasion factor. P21 pro-phagocytic activity may allow the parasite its intracellular permanence, where it can disrupt the phagolysosome compartment and grow freely in the host cell cytoplasm. Moreover, a well-known activity of a recombinant protein may turn out to be an alternative control tool for biologists to study processes possibly activated by another protein. For instance, the recombinant form of metacyclic trypomastigotes gp82 induces apoptotic cell death in melanoma cells [26]. We thus believe that P21 represents a novel tool for scientists to study phagocytosis.

All together, our results showed that P21-His_6_ upregulates phagocytosis and actin cytoskeleton remodeling by binding to CXCR4 receptor and signaling via PI3-kinase. Also, the recombinant protein activity sheds light on the possible role of the *T. cruzi* native P21 in regulating parasite escape from host immune response by giving the circulating parasites the chance to invade the host cell and multiply in the cytoplasm.

## Methods

### Reagents

PI3-kinase inhibitor, LY294002 (Cell Signaling), concentration: 50 µM, time of treatment: one hour prior to phagocytosis.

Ras – farnesyl thiosalicylic acid inhibitor (Cayman Chemical Company: 10010501), concentration: 50 µM, time of treatment: one hour prior to phagocytosis.

Akt inhibitor (Calbiochem: 124005), concentration: 25 µM, time of treatment: one hour prior to phagocytosis.

MEK 1/2 U0126 inhibitor (Cell Signaling: 9903), concentration: 10 µM, time of treatment: two hours prior to phagocytosis.

MEK 1 inhibitor PD98059 (Cell Signaling: 9900S), concentration: 50 µM, time of treatment: one hour prior to phagocytosis.

5-Iodotubercidin (ITU) (Santa Cruz Biotechnology: sc3531), concentration: 20 µM, time of treatment: one hour prior to phagocytosis.

Rapamycin (FRAP/mTOR inhibitor) and ERK2 inhibitor – (Cell Signaling), concentration: 10 nM, time of treatment: one hour prior to phagocytosis.

CXCR4 inhibitor - AMD 3100 octahydrochloride (MERCK), concentration: 30 µM, time of treatment: 30 minutes prior to phagocytosis.

TRITC-phalloidin -(Sigma-Aldrich: P5282), dilution: 1∶1500.

Polymyxin B (Sigma-Aldrich: 81271) (25 µg/ml). Time of treatment: 30 minutes prior to phagocytosis.

Bovine serum albumin (Sigma-Aldrich) was used as a control for P21-His_6_ at the same concentrations and time of incubation.

### Ethics

Maintenance and care of animals complied with the guidelines of the Laboratory Animal Ethics Committee from the Universidade Federal de Uberlândia. Animal euthanasia was performed in accordance with international welfare grounds, according to the American Veterinary Medical Association Guidelines on Euthanasia. This study was approved by the ethics committee of animal research from Universidade Federal de Uberlândia (process: CEUA/UFU 105/10).

### Animals

Male wild-type C57BL/6, *ccr4^−/−^* C3H/HePas and *toll-4^−/−^* mice were provided and maintained at the animal facilities of the Department of Biochemistry and Immunology, School of Medicine of Ribeirão Preto, USP (Ribeirão Preto, Brazil). Male mice were six to eight weeks old and were kept under standard conditions on a 12-h light-dark cycle in a temperature-controlled room (25±2°C) with food and water *ad libitum.*

### Parasite Culture

Promastigotes of *Leishmania* (*Leishmania*) *amazonensis* (IFLA/BR/67/PH8) were cultivated in Schneider’s Insect Medium (LGC Biotecnologia), supplemented with 10% fetal bovine serum (FBS; Cultilab, Campinas, Brazil), 100 µg/mL gentamicin and 2 mM L-glutamine (Gibco BRL-Life Technologies, New York, USA), at 26°C. Parasites in stationary phase were used for the experimental infection in macrophages. The parasites were maintained infective by periodic hamster footpad inoculations. Extracellular amastigotes from the *T. cruzi* G strain were obtained after differentiation of TCT in LIT medium, pH 5.8, overnight as previously described (Silva et al., 2006). Tachyzoites of the RH strain of *Toxoplasma gondii* were harvested from the peritoneal cavities of out-bred Swiss Webster mice that had been injected with 10^7^ organisms 2 days earlier.

### P21-His_6_ Purification

P21-His_6_ (GenBank: EU004210.1) was purified as previously described [12]. The quality of purification was demonstrated by SDS-PAGE.

### Peritoneal Macrophage Culture and Invasion Assays

Peritoneal macrophages from 6- to 8-week-oldmale C57BL/6 mice were harvested with 3 mL of Dulbecco’s modified Eagle’s medium (DMEM) from the peritoneal cavity of stimulated mice (1–3 mL of 3% thioglycollate medium; Gibco, Gaithersburg, MD; 3 days before) (mouse inflammatory macrophage). The cells were centrifuged and adjusted to 1×10^6^/mL in DMEM supplemented with 12.5 mM HEPES, 2 g/L sodium bicarbonate, 2 mM L-glutamine, 50 µg/mL gentamicin and 10% FBS (complete medium). The cells were seeded in 24-well flat-bottomed plates (Corning Corporation; Cambridge, MA, USA) at a density of 2×10^5^/200 µL/well and then incubated overnight in a humidified atmosphere with 5% CO_2_ at 37°C to allow the cells to adhere. Afterwards, 10 zymosan particles or 10 amastigotes of *T. cruzi* G strain or 20 promastigotes of *L. amazonensis* or 5 tachyzoites of *T. gondii* RH strain were added for each macrophage plated on a round coverslip in the presence or not of 40 µg/ml P21-His_6_. The parasites and zymosan particles were left in contact with macrophages for 2 hours and then washed with PBS, fixed in Bouin’s solution and Giemsa stained. In inhibitor- treated cells, treatment was performed according to the manufacturers’directions prior to the addition of zymosan particles. For the experiments with anti-P21-His_6_ polyclonal antibody, the recombinant protein was incubated or not with different dilutions of the polyclonal antibody for 30 minutes in DMEM and then used during zymosan phagocytosis assays using peritoneal macrophages with or without FcR blocking. For FcR blocking, serum samples from healthy C57BL/6 mice were diluted in DMEM (1∶20) and incubated with seeded macrophages for 30 minutes and then washed 3 times with PBS. Also, pre-immune serum samples were used as control for anti-P21-His_6_ antibody. Results were expressed as the number of internalized parasites or particles/100 cells.

### F-actin Staining

Inflammatory macrophages were recruited *in vivo* by intraperitoneal thioglycollate inoculation. After three days, cells were harvested from the mouse peritoneal cavity, washed 3 times with PBS, counted and adjusted to 1×10^6^ cells/mL in PBS. Macrophages with FcR blocking were pre-treated for 30 minutes with serum (1∶20 dilution) obtained from healthy C57BL/6 mice. The cells (FcR blocked or not) were then incubated with P21-His_6_, opsonized (anti-P21-His_6_) or not for different time periods: 30 minutes and one and three hours. The opsonized protein was obtained by incubating with serum of immunized C57BL/6 mice diluted 1∶10, 1∶40 and 1∶80, for 30 minutes. The cells were fixed with 4% formaldehyde, washed 3 times and then left in PBS. F-actin staining was performed using TRITC-phalloidin diluted 1∶1000 in PBS+saponin (0.01%). After washing, samples were analyzed by FACSCantoII (BD) and the results were obtained using FlowJo software (version 7.6.3).

### Western Blotting

To determine if the treatment of macrophages withP21-His_6_ would upregulate CXCR4 expression, 10^7^ peritoneal macrophages were seeded in a 6-well plate and treated or not with P21-His_6_for one hour. Following three PBS washes, macrophages were removed with a cell scraper and lysed in electrophoresis buffer (Santa Cruz Biotechnology). After SDS-PAGE and electrophoretic protein transfer, the membrane was incubated with anti-CXCR4 (Santa Cruz Biotechnology) followed by horseradish peroxidase-conjugated secondary antibody (Sigma-Aldrich). The CXCR4 band was detected by chemiluminescence, and densitometry was performed using ImageJ software. Similar amounts of spotted extracts were checked with anti-β-actin antibody (Sigma-Aldrich).

### Statistical Analysis

The significance of differences was determined by one-way ANOVA performed according to the VassarStats program (Richard Lowry 1998–2006), available at http://faculty.vassar.edu/lowry/VassarStats.html or using the GraphPad Prism program, version 5.01 for Student-t analysis. The differenceswere considered significant when p<0.05. All experiments were performed 4 to 8 times in triplicate.
